# The Efficacy of Accelerating Orthodontic Tooth Movement by Combining Self-Ligating Brackets With One or More Acceleration Methods: A Systematic Review

**DOI:** 10.7759/cureus.32879

**Published:** 2022-12-23

**Authors:** Heba M Al-Ibrahim, Mohammad Y Hajeer, Ahmad S Burhan, Kinda Sultan, Mowaffak A Ajaj, Luai Mahaini

**Affiliations:** 1 Department of Orthodontics, University of Damascus Faculty of Dentistry, Damascus, SYR

**Keywords:** surgically assisted tooth movement, vibrational forces, side effects, acceleration methods, self-ligating brackets, lllt, infrared light therapy, acceleration tooth movement, self-ligated brackets, orthodontics

## Abstract

This review aimed to evaluate the effectiveness of using one or more acceleration methods with self-ligating brackets to accelerate orthodontic tooth movement in adults and the associated effects of these interventions. An electronic search of the following databases (PubMed, Scopus, Google Scholar, EMBASE) was performed (From January 1990 to November 2021). ClinicalTrials.gov and the International Clinical Trials Registry Platform were also electronically searched to find any unpublished studies and ongoing trials. The selected randomized controlled trials (RCTs) involved adult patients treated using self-ligating brackets combined with one or more acceleration methods compared with self-ligating brackets or conditional brackets alone. The risk of bias was assessed using Cochrane’s risk of bias tool. A total of seven RCTs and one controlled clinical trial (CCT) were included in this review. Combining self-ligating brackets with flapless corticotomy, low-level laser therapy (LLLT), and infrared light accelerated orthodontic movement by 43% and 50% for surgical methods, 20-50% for LLLT, and 22% for infrared light. Regarding side effects on periodontal tissues, neither flapless corticotomy nor low-frequency vibrational forces caused any damage. Combining self-ligating brackets and flapless corticotomy, low-level laser, or infrared light effectively accelerated orthodontic movement by 20% to 50 %. In contrast, the combination of self-ligating brackets with vibrational forces did not affect speeding tooth movement. The acceleration methods did not have any side effects on the periodontal tissues, but the available evidence was insufficient. There is a need for further primary research regarding the effectiveness of combining self-ligating brackets with acceleration methods and the possible untoward side effects.

## Introduction and background

The use of self-ligating brackets in orthodontic treatment has become popular recently, which allows orthodontists to abandon the traditional means of archwires ligation (i.e., stainless steel ligatures and elastomeric modules) [1]. Depending on the locking mechanism, self-ligating brackets are classified into two main types: active self-ligating brackets (ASLBs), which lock tightly on the wire to express better rotation and torque values, and passive self-ligating brackets (PSLBs), which allow sliding movements [2]. An additional type of self-ligating brackets results from the combination of both active and passive brackets [3]. This type may result in greater efficiency in the expression of tip and torque values, faster movement of the anterior teeth, and lower loss of the anchorage [3]. There are many claims about the advantages of self-ligating brackets (SLBs) compared with conventional brackets (CBs) in dramatically reducing chair-side time, reducing the total treatment time, gaining better oral health, and causing less pain and discomfort [4].

The length of the period of orthodontic treatment has many disadvantages such as caries, periodontal diseases, patient complaints, fatigue, pain, and discomfort [5-9]. Therefore, many acceleration methods have been suggested in the medical literature to reduce the period of orthodontic treatment such as physical and biochemical methods (prostaglandin, calcium, laser, etc.) [10-12] and surgical procedures (corticotomy, corticision, and micro-osteoperforation, etc.) [13-20]. In addition, numerous studies have investigated the effect of self-ligating brackets in accelerating teeth movements [21-25]. Many have shown that using self-ligating brackets shortens the treatment duration [23-25].

Many systematic reviews have evaluated the effect of self-ligating brackets on the orthodontics movement, dimensions of the dental arches, and periodontal status [26-29]. However, no systematic review has assessed the participation between acceleration methods and self-ligating brackets on orthodontic movement and the associated effects.

Therefore, the current systematic review aims to identify the studies that have used acceleration methods in combination with self-ligating brackets and determine the effectiveness of these methods in orthodontic movement acceleration and their dentoalveolar effects.

## Review

Preliminary scoping search

Primarily, an exploratory search was carried out in the PubMed database. In the first stage of this review, the protocol was registered with PROSPERO (CRD42022367835), then the Cochrane Handbook for Systematic Reviews of Interventions and the Preferred Reporting Items for Systematic Reviews and Meta-Analyses (PRISMA) standards were followed in writing the current systematic review [30,31].

Eligibility criteria

The exclusion and inclusion criteria for the included studies in this systematic review were determined, and the PICOS (Population, Intervention, Comparison, Outcomes, and Study) framework was relied upon to identify participants, interventions, comparisons, outcomes, and study design. Regarding the targeted “population,” adult patients of any gender or ethnic group with any malocclusion who received orthodontic treatment with fixed orthodontic appliances. Concerning the “intervention,” any orthodontic treatment using fixed orthodontic appliances with self-ligating brackets assisted by one or more acceleration methods (surgical, physical, biological, etc.) was involved. The “comparison” group should include patients receiving orthodontic treatment with fixed appliances using self-ligating or conventional brackets alone (without any acceleration method). The “outcomes” of interest were the rate of orthodontic tooth movement or any equivalent measurement that would determine the effectiveness of the acceleration procedures, dentoalveolar changes, and periodontal status, including loss of attachment, gingival recession, depth of probing, bone resorption, loss of attachment, or teeth damage following the orthodontics movements (e.g., root resorption).

Search strategy

The search was accomplished electronically from January 1, 1990, to November 2021 by two independent reviewers (HMI, MYH) without regard to time or language using the following databases: PubMed, Scopus®, EMBASE®, and Google Scholar. The search strategy is described in detail in Table 1. For any potential concerning studies that the search on the web may not have observed, the chosen papers' reference lists and the related reviews were checked. To explore unpublished articles or research works, World Health Organization International Clinical Trials Registry Platform Search Portal (ICTRP) and ClinicalTrials.gov were also investigated.

**Table 1:** Electronic search strategy

Electronic database	Search strategy
PubMed	#1 (Self-ligated brackets “OR“ Self-ligating brackets “OR“ Self-ligating braces “OR” Self-ligated braces “OR “ Orthodontic self-ligation “OR” Orthodontic Self-ligated “OR” Orthodontic Self-ligating “OR“ SLBs) #2 (Orthodontics tooth movement “OR” Accelerate “OR” Acceleration “OR” Rate of tooth movement “OR” Rapid tooth movement “OR” Accelerated tooth movement “OR” Acceleration of tooth movement “OR” Speed tooth movement “OR” Rapid orthodontic tooth movement “OR” Orthodontic treatment time “OR” Duration of orthodontic treatment “OR” Short treatment time “OR” Reduction treatment time “OR” Rapid Orthodontics “OR” Accelerated orthodontics “OR” Accelerated orthodontic treatment “OR“ Regional accelerated phenomenon “OR” RAP) #3 (Cytokine ”OR“ vitamin D ”OR“ RANKL ”OR“ RANK ”OR“ MCSF ”OR“ PTH ”OR“ OPG ”OR“ Prostaglandins “OR” Calcium “OR” Photobiomodulation “OR” Vibrational stimulation “OR” Resonance “OR” Vibration “OR” Direct electrical current “OR” Light-Emitting Diode “OR” Surgical assisted tooth movement “OR” segmental alveolar distraction “OR” Intraseptal alveolar surgery “OR” soft lasers “OR” Double irradiation “OR” Single irradiation ”OR “ LLLT ” OR “ Low-Level Laser Therapy “OR” Light-emitting diode “OR” Micro-incisions OR Microincisions “OR” Micro-osteoperforations “OR” corticotomy “OR” alveolar decortication “OR” Selective alveolar decortication “OR” Corticision “OR“ Corticopuncture “OR” Cortico-puncture “OR” Piezoelectric surgery “OR” piezoelectric “OR” Piezosurgery “OR”piezocision “OR” Piezopuncture “OR” Piezotome-Corticision Assisted Orthodontics “OR” Piezoelectric Corticotomies “OR” Piezocision-assisted orthodontic treatment.” #4 #1 AND #2 AND #3
Scopus	#1 TITLE-ABS-KEY (Self-ligated brackets “OR“ Self-ligating brackets “OR“ Self-ligating braces “OR” Self-ligated braces “OR “ Orthodontic self-ligation “OR” Orthodontic Self-ligated “OR” Orthodontic Self-ligating “OR“ SLBs) #2 TITLE-ABS-KEY (Orthodontics tooth movement “OR” Accelerate “OR” Acceleration “OR” Rate of tooth movement “OR” Rapid tooth movement “OR” Accelerated tooth movement “OR” Acceleration of tooth movement “OR” Speed tooth movement “OR” Rapid orthodontic tooth movement “OR” Orthodontic treatment time “OR” Duration of orthodontic treatment “OR” Short treatment time “OR” Reduction treatment time “OR” Rapid Orthodontics “OR” Accelerated orthodontics “OR” Accelerated orthodontic treatment “OR“ Regional accelerated phenomenon “OR” RAP) #3 TITLE-ABS-KEY (Cytokine ”OR“ vitamin D ”OR“ RANKL ”OR“ RANK ”OR“ MCSF ”OR“ PTH ”OR“ OPG ”OR“ Prostaglandins “OR” Calcium “OR” Photobiomodulation “OR” Vibrational stimulation “OR” Resonance “OR” Vibration “OR” Direct electrical current “OR” Light-Emitting Diode “OR” Surgical assisted tooth movement “OR” segmental alveolar distraction “OR” Intraseptal alveolar surgery “OR” soft lasers “OR” Double irradiation “OR” Single irradiation ”OR “ LLLT ” OR “ Low-Level Laser Therapy “OR” Light-emitting diode “OR” Micro-incisions OR Microincisions “OR” Micro-osteoperforations “OR” corticotomy “OR” alveolar decortication “OR” Selective alveolar decortication “OR” Corticision “OR“ Corticopuncture “OR” Cortico-puncture “OR” Piezoelectric surgery “OR” piezoelectric “OR” Piezosurgery “OR”piezocision “OR” Piezopuncture “OR” Piezotome-Corticision Assisted Orthodontics “OR” Piezoelectric Corticotomies “OR” Piezocision-assisted orthodontic treatment” #4 #1 AND #2 AND #3
Embase	#1 (Self-ligated brackets “OR“ Self-ligating brackets “OR“ Self-ligating braces “OR” Self-ligated braces “OR “ Orthodontic self ligation “OR” Orthodontic Self-ligated “OR” Orthodontic Self-ligating “OR“ SLBs) #2 (Orthodontics tooth movement “OR” Accelerate “OR” Acceleration “OR” Rate of tooth movement “OR” Rapid tooth movement “OR” Accelerated tooth movement “OR” Acceleration of tooth movement “OR” Speed tooth movement “OR” Rapid orthodontic tooth movement “OR” Orthodontic treatment time “OR” Duration of orthodontic treatment “OR” Short treatment time “OR” Reduction treatment time “OR” Rapid Orthodontics “OR” Accelerated orthodontics “OR” Accelerated orthodontic treatment “OR“ Regional accelerated phenomenon “OR” RAP) #3 (Cytokine ”OR“ vitamin D ”OR“ RANKL ”OR“ RANK ”OR“ MCSF ”OR“ PTH ”OR“ OPG ”OR“ Prostaglandins “OR” Calcium “OR” Photobiomodulation “OR” Vibrational stimulation “OR” Resonance “OR” Vibration “OR” Direct electrical current “OR” Light-Emitting Diode “OR” Surgical assisted tooth movement “OR” segmental alveolar distraction “OR” Intraseptal alveolar surgery “OR” soft lasers “OR” Double irradiation “OR” Single irradiation ”OR “ LLLT ” OR “ Low-Level Laser Therapy “OR” Light-emitting diode “OR” Micro-incisions OR Microincisions “OR” Micro-osteoperforations “OR” corticotomy “OR” alveolar decortication “OR” Selective alveolar decortication “OR” Corticision “OR“ Corticopuncture “OR” Cortico-puncture “OR” Piezoelectric surgery “OR” piezoelectric “OR” Piezosurgery “OR”piezocision “OR” Piezopuncture “OR” Piezotome-Corticision Assisted Orthodontics “OR” Piezoelectric Corticotomies “OR” Piezocision-assisted orthodontic treatment.” #4 #1 AND #2 AND #3
Google Scholar	(Self-ligated brackets “OR“ Self-ligating brackets “OR“ Self-ligating braces “OR” Self-ligated braces “OR “ Orthodontic self-ligation “OR” Orthodontic Self-ligated “OR” Orthodontic Self-ligating “OR“ SLBs) AND (Orthodontics tooth movement “OR” Accelerate “OR” Acceleration “OR” Rate of tooth movement “OR” Rapid tooth movement “OR” Accelerated tooth movement “OR” Acceleration of tooth movement “OR” Speed tooth movement “OR” Rapid orthodontic tooth movement “OR” Orthodontic treatment time “OR” Duration of orthodontic treatment “OR” Short treatment time “OR” Reduction treatment time “OR” Rapid Orthodontics “OR” Accelerated orthodontics “OR” Accelerated orthodontic treatment “OR“ Regional accelerated phenomenon “OR” RAP) AND (Cytokine ”OR“ vitamin D ”OR“ RANKL ”OR“ RANK ”OR“ MCSF ”OR“ PTH ”OR“ OPG ”OR“ Prostaglandins “OR” Calcium “OR” Photobiomodulation “OR” Vibrational stimulation “OR” Resonance “OR” Vibration “OR” Direct electrical current “OR” Light-Emitting Diode “OR” Surgical assisted tooth movement “OR” segmental alveolar distraction “OR” Intraseptal alveolar surgery “OR” soft lasers “OR” Double irradiation “OR” Single irradiation ”OR “ LLLT ” OR “ Low Level-Laser Therapy “OR” Light-emitting diode “OR” Micro-incisions OR Microincisions “OR” Micro-osteoperforations “OR” corticotomy “OR” alveolar decortication “OR” Selective alveolar decortication “OR” Corticision “OR“ Corticopuncture “OR” Cortico-puncture “OR” Piezoelectric surgery “OR” piezoelectric “OR” Piezosurgery “OR”piezocision “OR” Piezopuncture “OR” Piezotome-Corticision Assisted Orthodontics “OR” Piezoelectric Corticotomies “OR” Piezocision-assisted orthodontic treatment”

Study selection and data extraction

The eligibility of the trials was estimated by two reviewers (HMA and MYH) separately, and if there were any odds, a third author (EZ) was asked to settle this. First, the titles and abstracts only were checked. Full-text estimation as a second step for all relevant papers and elected for implication. Full-text estimation was also done when the titles or abstracts were not obvious. The documents which did not conform to one or more of the inclusion criteria were kept out. For any elucidation or extra details, the corresponding authors were e-mailed.

The two authors (HMA and MYH) extracted data independently in the predefined tables for extracted data. To resolve any disagreements between the two authors, a third author (EZ) was consulted. The following elements were listed on the data extraction sheet: basic information (authors' names, publication year, and study location); methodology (research design: either a split-mouth design (SMD) or a parallel-group design (PGD), comparison of several treatments); participants (age, gender, sample size); intervention (location, type, technical characteristics); orthodontic consideration (malocclusion types, features, and biomechanics of applied devices, follow-up duration); and outcomes (primary and secondary outcomes mentioned, measurement methods, statistical significance of differences between experimental and control groups).

Assessment of risk of bias in included studies and strength of evidence

Using Cochrane’s risk of bias tool, the two reviewers (HMA and MYH) evaluated the quality of the included studies [32]. If there were any disagreements, a third author (ASB) was asked to make a final decision. The risk of bias for the following fields was rated as low, high, or unclear: sequence generation (selection bias), allocation concealment (selection bias), blinding of participants and personnel (performance bias), blinding of outcome assessors (detection bias), incomplete outcome data addressed (attrition bias), selective outcome reporting (reporting bias), and other bias.

The following criteria were used to determine how likely each trial was to be biased overall: if all fields were considered to have a low risk of bias, the trial would be viewed at a low risk of bias where bias is unlikely to alter the outcomes significantly; if one domain at least or more were determined to have an uncertain risk of bias, the trial would be deemed to have an unclear risk of bias, which releases some doubt on the outcomes; and if one domain at least or more were decided to have a high risk of bias, the trial would be assessed at high risk of bias where bias impact the outcomes critically.

Results

Study Selection and Inclusion in the Review

**Figure 1:**
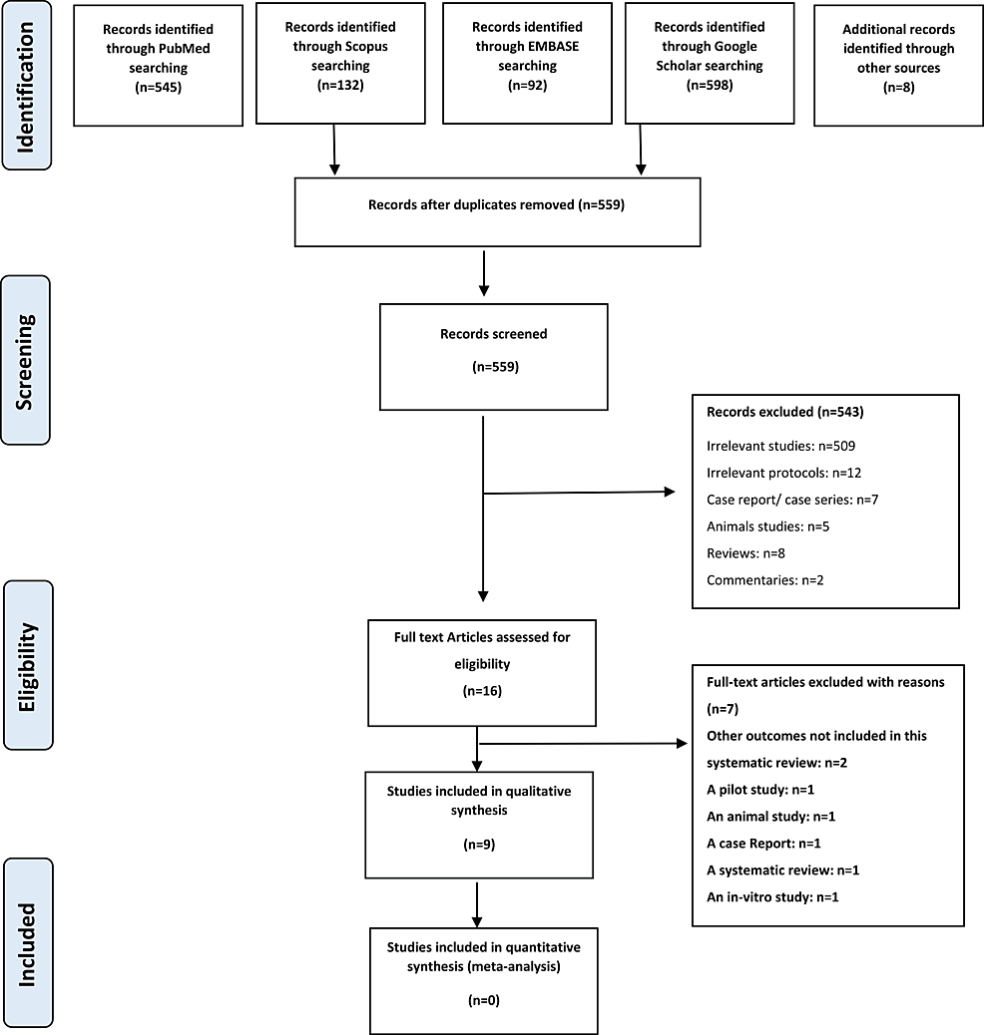
The Preferred Reporting Items for Systematic Reviews and Meta-Analyses (PRISMA) flow diagram of study identification, screening, and inclusion in this review

In the electronic search, 1367 references were discovered and eight more records were identified from other sources. Repeated references were omitted then 559 citations were carefully checked. A comprehensive screening of the titles and abstracts was accomplished for eligibility, and the papers not fulfilling the inclusion criteria were discarded. As a result, 12 trials were likely related and were thoroughly reviewed. Following the full-text reading of the publications, four studies were eliminated (Table 2). Ultimately, seven RCTs and one CCT were included. The PRISMA flow diagram is shown in Figure 1.

**Table 2:** Excluded studies and the reasons beyond exclusion

Study	Reason for exclusion
Caccianiga, G., Cordasco, G., Leonida, A., Zorzella, P., Squarzoni, N., Carinci, F., & Crestale, C. (2012). Periodontal effects with self-ligating appliances and laser biostimulation. Dental research journal, 9(Suppl 2), S186.	This study aimed to examine the effects of low-energy laser irradiation on stimulating keratinized gingiva in patients with teeth that erupted in oral mucosa completely, not the effect of the participation of self-ligating brackets with acceleration methods on orthodontic movement.
Caccianiga, G., Stanizzi, A., Zorzella, P., Crestale, C., Denotti, D., & Squarzoni, N. (2012). Laser Biostimulation and Self Ligating Appliances in Orthodontics: Periodontal Remodeling. European Journal of Inflammation, 10(2_suppl), 55-59.	This study aimed to examine the combination between self-ligating appliances and laser biostimulation in promoting attached gingiva around the crown of the teeth erupted in oral vestibular mucosae not on orthodontic movement.
Teng, G. Y., & Liou, E. J. (2014). Interdental osteotomies induce regional acceleratory phenomena and accelerate orthodontic tooth movement. Journal of Oral and Maxillofacial Surgery, 72(1), 19-29.	An animal study: the maxillary incisors, from the right third incisor to the left third incisor, were bonded in an alternate sequence of self-ligating brackets (Damon System) and conventional brackets (OPAK) in the sham control an experimental group both. The experimental group received orthodontic tooth alignment of the maxillary incisors and interdental osteotomies between the maxillary third incisor and canine on both sides.
Mittal, R., Attri, S., Batra, P., Sonar, S., Sharma, K., & Raghavan, S. (2020). Comparison of orthodontic space closure using micro-osteoperforation and passive self-ligating appliances or conventional fixed appliances: A randomized controlled trial. The Angle Orthodontist, 90(5), 634-639.	This study aimed to compare the effect of using self-ligating brackets or conventional brackets with micro-osteoperforation (MOP) on the space closure rate without the existence of the control group (self-ligating brackets alone/conventional brackets alone).

Characteristics of Studies

The characteristics of the eight included studies are mentioned in Table 3. The studies included in this systematic review consist of seven RCTs [25,33-39] and one controlled clinical trial (CCT) [40]. The total number of patients included in all studies was 339, aged between 12.9 and 34 years. Six studies included both genders [25,33,34,36-39] while two studies did not report any information about sex distribution [35,40]. Seven of the included trials were of a parallel-group design (PGD) [25,33-35,37-40], and only one trial was of a split-mouth design (SMD) [36]. All studies selected were based on evaluating the effectiveness of the combination of SLBs with one of the acceleration methods. Two trials investigated SLBs + flapless corticotomy [25,33], one trial studied SLBs + infrared light therapy [35], three studies examined SLBs + low-level laser (LLT) [36,38,40], and two studies evaluated SLBs + low-frequency mechanical vibrations [34,37].

**Table 3:** Characteristics of the included studies M: Male, F: Female, RCT: Randomized clinical trial, PGD: Parallel-group design, SLBs: Self-ligating brackets, Exp: Experimental, mW/cm2: Milliwatts per centimeter squared, LED: Light-emitting diode, SMD: Split-mouth design, LLT: Low-intensity laser therapy, nm: nanometer, RTM: Rate of tooth movement, CBs: Conventional brackets, VA: Vibrational appliance, IL-1β: Interleukin-1 beta, GCF: Gingival crevicular fluid, PD: Periodontal depth, LFV: Low-frequency vibrations, Hz: Hertz, mm: Millimeter, CCT: Controlled clinical trial, LILT: Low-intensity laser therapy, J/cm2: Joule per cm2, J: Joule, FC: Flapless corticotomy

Study	Methods		Participants	Type of Malocclusion	Interventions		Outcomes
Authors, year of publication, and country	Study design	Treatment comparison	Patients (M/F), Age (years)		Type and site of intervention/technical aspects of interventions	Follow-up time	Primary and secondary outcomes
Charavet et al. 2016 Belgium [33]	RCT (PGD)	(SLBs + Piezocision) vs. SLBs	Patients (M/F): 24 (9/15) Control: 12, Exp: 12 Mean age (years): Control: 27± 7 Exp: 34±8	Patients with minimal to moderate maxillary and mandibular anterior crowding (non-extraction treatment plan)	Vertical interproximal microincisions were created below each interdental papilla	Until complete the overall orthodontic treatments	Primary outcome: the treatment time (days) Secondary outcomes: - periodontal health - alveolar crest changes - one and gingival healing - analgesic intake - patient-centered outcomes
Nahas et al. 2016 United Arab Emirates [35]	RCT (PGD)	(SLBs + Infra-red light) vs. SLBs	Patients: 40 Control: 20, Exp: 20 Mean age (years): Control: 21.1±10.2 Exp:21.8±5.3	Patients with lower anterior crowding (non-extraction treatment plan)	Irradiation of the lower anterior segment at a wavelength of 850 nm and a power output of 90 mW/cm2 for 20 min daily using an extraoral LED device	Until completing the leveling and aligning phase	Treatment time
Qamruddin et al. 2017 Pakistan [36]	RCT (SMD)	(SLBs + LLT) vs. SLBs	Patients (M/F): 22 (11/11) Mean age (years): 19.8±3.1	Patients with Angle Class II Division 1 malocclusion (required extraction of maxillary first premolars bilaterally)	A gallium-aluminum-arsenic diode laser with a wavelength of 940 nm in a continuous mode was applied at 5 points buccally and palatally around the canine roots on the experimental side. Laser irradiation was applied at baseline and then repeated after 3 weeks for 2 more consecutive follow-up visits.	Every 3 weeks for 3 more consecutive visits	Primary outcome: RTM (mm/3 weeks) Secondary outcomes: pain
Kalemaj et al. 2017 Italy [34]	RCT (PGD)	CBs vs. SLBs vs. (SLBs+ VA)	Patients (M/F): 33 (14/19), CBs: 11, SLBs: 11 SLBs + VA: 11, Mean age (years): CBs: 12.9 ± 1.85, SLBs: 13.3 ± 2.8, SLBs + VA: 13.1 ± 0.07	Patients with lower anterior crowding (non-extraction treatment plan)	Patients were instructed to use the vibratory device (AccleDent) for 20 minutes daily, beginning from the day of appliance placement and continuing for the first 4 weeks.	The first 3 months of the leveling and aligning phase	Primary outcome: Rate of mandibular incisors alignment Concentration of IL-1β Pain and discomfort Secondary outcomes: Quantity of GCF PD
Lalnunpuii et al. 2020 India [38]	RCT (PGD)	(SLBs + LLT) vs. (CBs + LLT) vs. CBs	Patients (M/F): 65 (24/41), SLBs + LLT: 20, CBs + LLT: 20, CBs: 25, Mean age (years): SLBs + LLT: 17.9 ± 1.9, CBs + LLT: 17.9 ± 1.9, CBs: 17.5 ± 1.3	Patients who need to extract maxillary 1st premolars and en-mass retraction	The low-level laser was applied in the laser groups using a 658 nm (Aluminum, Gallium, Arsenide) semiconductor diode laser. Two irradiations were done both buccally and palatally/lingually from canine to canine. The laser regimen was applied on days 0, 3, 7, and 14 in the first month, then on every 15th day until complete en-masse retraction.	Until complete en-masse retraction	The rate of space closure (mm/months)
Kumar et al. 2020 India [37]	RCT (PGD)	(SLBs + LFV) vs. (CBs + LFV) vs. CBs	Patients (M/F): 65 (30/35) SLBs + LFV: 20 CBs + LFV: 20 CBs: 25, Mean age (years): SLBs + LFV: 17 ± 0.80, CBs + LFV: 17.40 ± 0.72, CBs: 16.90 ± 1.1	Patients who need to extract maxillary 1st premolars and en-mass retraction	The low-frequency vibrations were provided by a custom-made vibratory device. The device was used for 20 minutes daily (during the space closure phase) at a frequency of 30 Hz.	Until completing the space closure	The rate of space closure (mm/months)
Chandran et al. 2020 India [40]	CCT (PGD)	Group IA (CBs+ LILT), vs. Group IB (CBs) vs. Group IIA (SLBs+ LILT) vs. Group IIb (SLBs)	Patients (M/F): 32 (NR) Group IA: 8, Group IB: 8, Group IIA: 8, Group IIB: 8, Mean age (years): 19.15 ± 2.26	Patients with lower anterior crowding (non-extraction treatment plan)	Photobiomodulation with LILT. Gallium Aluminum Arsenide (GaAlAs) diode laser of 808 nm wavelength in a continuous wave of 8 J/cm2 and energy of 2 J per point was used. Laser irradiation was done on the 0, 3rd, 7th, and 14th days in the first month and in intervals of 15 days from the second month until the complete alignment was achieved.	Until completing the leveling and aligning phase	The time taken for the decrowding of the lower anterior teeth (days)
Al-Ibrahim et al. 2021 Syria [25]	RCT (PGD)	(SLBs + FC) vs. SLBs vs. CBs	Patients (M/F): 58 (10/47) SLBs + FC: 19 SLBs: 19 CBs: 19 Mean age (years): SLBs + FC: 20.67 ± 2.59, SLBs: 19.98 ± 2.84, CBs: 19.62 ± 2.42	patients with severe upper crowding who need to extract maxillary 1st premolars.	Incisions 5 to 8 mm long and 3 mm deep were performed 4 mm beneath the papilla. Corticotomies were done only once for each patient at the start of treatment.	Until completing the leveling and aligning phase	Primary outcome: Leveling and alignment time, Secondary outcomes: Periodontal assessment

Four studies involved non-extraction‑based treatments [33-35,40], and four involved extraction-based treatments [25,36-39]. All extraction-based studies required the extraction of maxillary first premolars [25,36-38] except for one study, which involved the extraction of the first premolars in both arches [39]. Five trials focused on anterior crowding [25,33-35,40], where three of them involved anterior crowding on the lower jaw only [34,35,40], one on the upper jaw only [25], and one on the two jaws [33]. All crowding-based studies did involve teeth extraction to relieve crowding [33-35,40], except for one study [25]. Three papers studied Class II division 1 [36-38], which was treated using en-mass retraction in two [37,38]. For the secondary outcomes, three studies assessed the periodontal status [25,33,34], whereas no trial evaluated the dentoalveolar changes.

Risk of Bias of Included Studies

**Figure 2:**
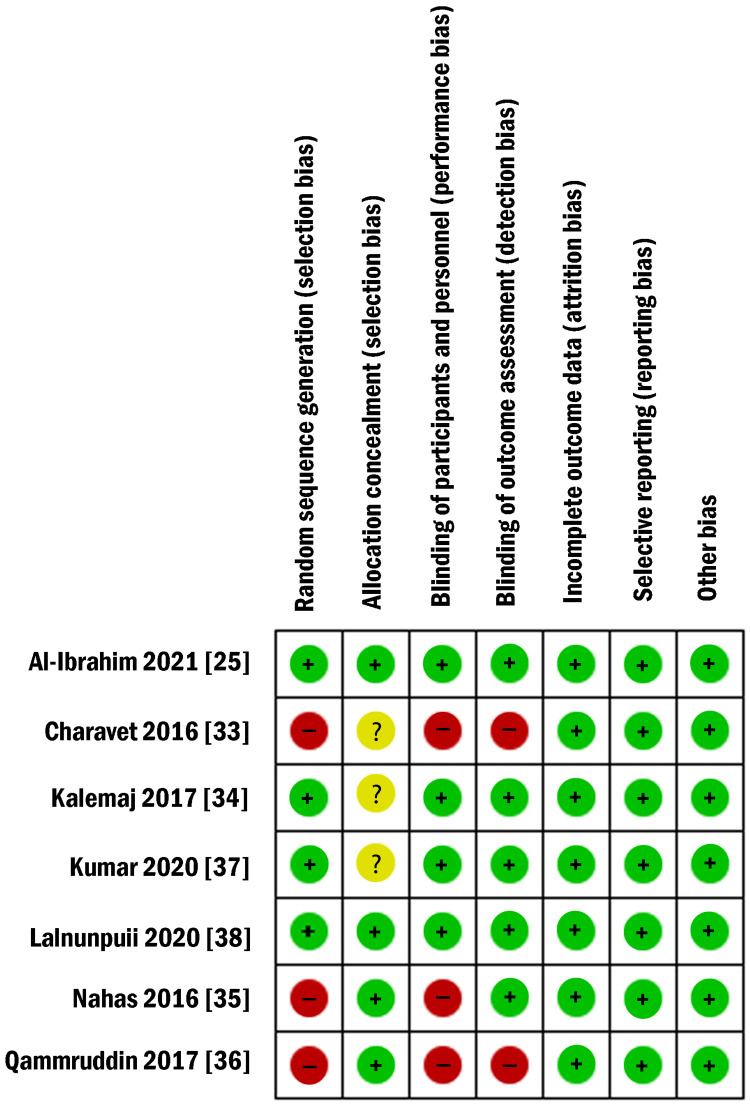
Risk of bias summary: review authors’ judgments about each risk of bias item for each included study

**Figure 3:**
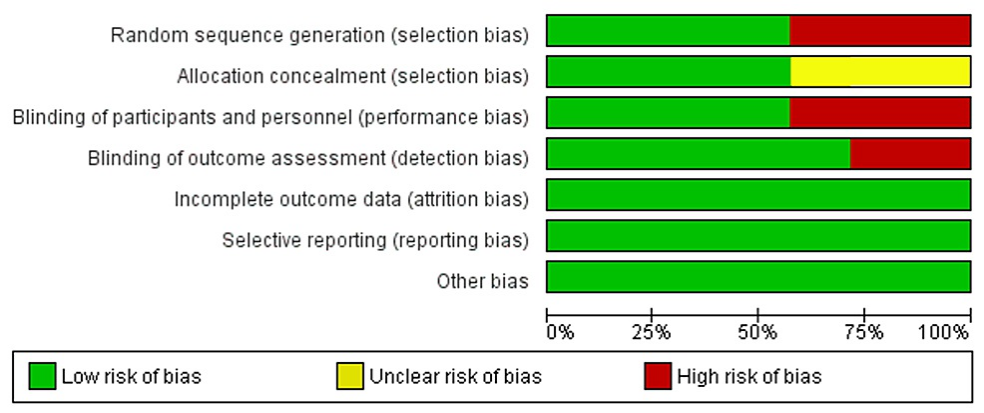
The overall risk of bias score for each field of randomized controlled trials

The summary and the overall risk of bias for the enclosed RCTs are depicted in Figures 2, 3. Table 4 shows the risk of bias for the non-randomized study according to the MINORS (Methodological Index for Non-Randomized Studies) tool. Two RCTs were classified as ‘low risk of bias’ [25,38]. Two RCTs were judged to be at an 'unclear risk of bias' [34,37] while the three others were assessed as having a 'high risk of bias' [33,35,36]. The random sequence generation and the participants’ blinding were evaluated as a 'high risk of bias' in three RCTs, and the blinding of outcomes assessors was assessed as a 'high risk of bias' in two RCTs. The allocation concealment was unclear in three RCTs. More information about the risk of bias can be seen in Table 5. For the only CCT included [40], according to the MINORS tool, the most problematic domains were the inclusion of consecutive patients and prospective calculation of the study sample size. The risk of bias score was 19/24, which meant fair quality.

**Table 4:** Risk of bias for non-randomized studies according to the related risk of bias tool

Reference	1. A clearly stated aim	2. Inclusion of consecutive patients	3. Prospective collection of data	4. Endpoints appropriate to the aim of the study	5. Unbiased assessment of the study endpoint	6. Follow-up period appropriate to the aim of the study	7. Loss to follow-up less than 5%	8. Prospective calculation of the study size	9. An adequate control group	10. Contemporary groups	11. Baseline equivalence of groups	12. Adequate statistical analyses	Total
Chandran et al. 2020 [40]	2	0	2	2	2	2	2	0	2	2	2	1	19/24

**Table 5:** Explanation of each judgment for each domain in the risk of bias assessment of the included randomized controlled trials CT: Computed tomography, LED: Light-emitting diode, LLLI: Low-level laser irradiation, GCF: Gingival crevicular fluid

Study	Sequence generation	Allocation concealment	Blinding of participants and personnel	Blinding of outcome assessment	Incomplete outcome data	Selective reporting	Other bias
Charavet et al. 2016 Belgium [33]	High risk: No mention of the method used for randomization.	Unclear risk: No mention of the method used to conceal the allocation sequence. “Twenty-four adult patients presenting with mild overcrowdings were randomly allocated to either a control group that was treated with conventional orthodontics or a test group that received piezo-assisted orthodontics.”	High risk: No mention of this point.	High risk: No mention of this point.	Low risk: “All patients were followed until the completion of treatment. Two patients (1 in each group) failed to show up for the post-treatment CT scan and were excluded from the follow-up.” incomplete outcome data adequately. Addressed (number, reasons)	Low risk: The protocol for the study was registered in clinical trial.gov study ID: NCT03406130 and the outcomes that mentioned in the protocol have been reported (unless using CT instead of CBCT).	Low risk: No other forms of bias seemed to be found.
Nahas et al. 2016 United Arab Emirates [35]	High risk: No mention of the method used for randomization.	Low risk: “The patients were randomly assigned to one of the two groups utilizing simple randomization by asking them to draw a sealed envelope (n = 40) that indicated their allocation to a test group (n = 20) in which LED.	High risk: No mention of this point.	Low risk: “The alignment of the lower six anterior teeth was evaluated by a single investigator who was blinded regarding the patients’ group allocations.”.	Low risk: “a total of four patients were dropped out from the control group, three required interproximal reduction of the lower anterior teeth, and one failed to attend more than three consecutive appointments. In the test group, two patients were excluded from the study; the first patient did not commit to using the LED device with a compliance rate of above 80 % and the second patient experienced a malfunction with his LED device “ incomplete outcome data adequately. Addressed (number, reasons)	Low risk: The protocol was not registered. But the reported outcomes in the result section seemed to be corresponding with the pre-defined outcomes aforesaid in the method section.	Low risk: No other forms of bias seemed to be found.
Qammruddin et al. 2017 Pakistan [36]	High risk: No mention of the method used for randomization.	Low risk: “The maxillary arch was divided into experimental and placebo groups randomly by ﬂipping a coin. LLLI was”.	High risk: No details of blinding of participants or personnel.	High risk: No details of blinding of outcome assessors.	Low risk: “Twenty-two patients were recruited for the study, of whom 2 were dropped because of dislodgement of the coil spring prematurely in 1 patient and analgesics taken by the other." (incomplete outcome data adequately addressed (numbers, reasons )	Low risk: The protocol was not registered. However, the reported outcomes in the result section seemed to be corresponding with the pre-defined outcomes aforesaid in the method section.	Low risk: No other forms of bias seemed to be found.
Kalemaj et al. 2017 Italy [34]	Low risk: “A list of block randomization with variable block size scheme of 3 and 6 was generated on Stata using the command “-ralloc” (StataCorp, College Station, Tex).”	Unclear risk: “Allocation concealment was obtained implementing a centralized assignment which did not involve trial investigators and staff.”	Low risk: “Blinding of patients and practitioner was not feasible”	Low risk: “however outcome assessment was blind because the GCF samples, models, and questionnaires were enumerated in a sequential order from the first to the last collection with no reference to pertaining patients or groups.”	Low risk: “No patient was excluded from the study and there were no lost to follow-up.”	Low risk: The protocol was not registered. But the reported outcomes in the result section seemed to be corresponding with the pre-defined outcomes aforesaid in the method section.	Low risk: No other forms of bias seemed to be found.
Lalnunpuii et al. 2020 India [38]	Low risk: “Randomization was carried out, using a computer-generated random allocation sequence to ensure equivalent distribution amongst the 3 groups.”	Low risk: “the sequences were concealed and were chosen by the patient.”	Low risk: “Blinding of the participants and the primary investigator was not possible due to the nature of the trial.”	Low risk: “Only the data analyser was blinded to the groups and the digital models presented to the data analyser were also coded.”	Low risk: The consort flow chart of participants through each stage of the trial shows there was no loss of participants.	Low risk: “the trial is registered at the National Trial Registry (CTRI/2018/04/013156)”	Low risk: No other forms of bias seemed to be found.
Kumar et al. 2020 India [37]	Low risk: “Randomization was carried out using a computer-generated random allocation sequence.”	Unclear risk: “The sequences were concealed and were chosen by the patient.”	Low risk: “Blinding of the participants and the primary investigator was not possible due to the nature of the trial”	Low risk: “Only the data analyser was blinded to the groups and the digital models presented were coded”	Low risk: “There was neither any loss of participants for any group nor a reported malfunction by any patient for the vibratory device”	Low risk: “the trial is registered at the National Trial Registry (CTRI/2018/04/013009).”	Low risk: No other forms of bias seemed to be found.
Al-Ibrahim et al. 2021 Syria [25]	Low risk: " SPSS for Windows, version 20 (IBM Corporation, Armonk, NY, USA) was used to determine the allocation of patients among the 3 groups, using a set of random numbers with an allocation ratio of 1:1:1.”	Low risk: “The allocation sequence was hidden using numbered, covered, and closed envelopes, which were opened only after performing all patients’ assessments and premolar extractions.”	Low risk: “It was not possible to make the treatment procedures blind for either patients or practitioners”	Low risk: ”so blinding was limited to data analysis”	Low risk: “No patient was lost to follow-up; therefore, 57 patients were included in the data analysis”	Low risk: “The current trial was registered at ClinicalTrials.gov (ID: NCT04950829)”	Low risk: No other forms of bias seemed to be found.

Effects of Interventions: Primary Outcome: Rate of Tooth Movement

Out of the eight studies, two studies only assessed the influence of combining surgical methods (using flapless corticotomy in particular) with SLBs [25,33], and the other six studies evaluated combining physical methods with SLBs [34-38,40]. Three of those six studies included LLLT with SLBs [36,38,40], one involved infrared light [35], and the other two studies assessed low-frequency vibrations with SLBs [34,37].

Combining flapless corticotomy with SLBs: Two parallel-group design studies evaluated the effectiveness of combined SLBs with piezocision [25,33]. Charavet et al. conducted their study on 24 adult patients with mild to moderate crowding, where they found a 43% decrease in total treatment time when using SLBs with piezocision compared to the traditional orthodontic treatment [33]. As for the study of Al-Ibrahim et al. on 58 patients with severe crowding on the upper jaw, it was shown that the participation of self-ligating brackets with the flapless corticotomy contributed to an acceleration of orthodontic movement by 50% [25].

Combining low-level laser therapy (LLLT) with SLBs: There are one split-mouth design study [36] and two parallel-group design studies evaluated using the low-level laser with self-ligating brackets [38,40]. Qamruddin and his colleagues experimented on 20 patients with a Class II division 1 malocclusion using the split-mouth design [36]. They found that the retraction of the canines on the experimental side was greater compared to the control group (1.6 mm/month vs. 0.79 mm/month, respectively) [36].

For the two parallel-group design studies, Lalnunpuii et al.'s study focused on the investigation of en-mass retraction in young patients and concluded that the rate of orthodontic movement was faster in the experimental group compared to the control group (≃ 0.66 mm/month vs. 0.48 mm/month; respectively) [38]. The other study focused on assessing decrowding in adult patients, where it found that the combination of SLBs with the LLLT accelerated the correction of lower crowding by 20% [40].

Combining low-frequency vibrations with SLBs: For this field, two studies of three-arm design were found [34,37]. One study experimented on a group of adult patients who had crowding on the lower incisors without the need for extraction [34], and the other study was on young patients who underwent en-mass retraction [37]. Both studies did not find any effect of the low-frequency vibrational forces on accelerating the rate of orthodontic tooth movement.

Combining infrared light with SLBs: There is only one trial with a parallel-group design conducted by Nahas and his colleagues [35]. They investigated using self-ligating brackets with extraoral infrared light for 20 minutes/day compared to a control group [35]. They conducted their study on 20 patients with moderate dental crowding cases and concluded that the participation of self-ligating brackets with infrared light contributed to the acceleration of leveling and alignment by 22% [35].

The Secondary Outcomes: The Periodontal Status

Three studies examined the periodontal status associated with the participation of one of the acceleration methods with self-ligating brackets [25,33,34]. In Charavet's study on 24 adult patients who needed decrowding on both jaws without extraction, they found no gingival recession following piezocision with SLBs [33]. Kalemaj et al. found no negative effects on periodontal depth after applying low-frequency vibrational forces with SLBs [34]. Furthermore, Al-Ibrahim et al. found that applying flapless corticotomy with self-ligating brackets had no adverse effects on periodontal indices [25].

Discussion

This systematic review is the first in the literature to evaluate the efficiency of the combination of self-ligating brackets with different acceleration methods in various malocclusion cases. The current systematic review performed a qualitative evaluation including 339 patients in eight selected clinical studies.

To reduce confounding factors, the selection of studies was confined to randomized and non-randomized controlled clinical trials only. A quarter of the included studies were rated at low risk of bias. On the other hand, the blinding of patients and the random sequence generation were considered the most problematic fields in the other studies.

The two studies that evaluated the combination of self-ligating brackets with surgical methods using flapless corticotomy showed an acceleration of 43% and 50% [25,33]. The acceleration obtained in these two studies can be explained by the effect of participating in the regional acceleratory phenomenon (RAP) with the mechanical effect of self-ligating brackets [25,33]. On the other hand, the difference in the rate of acceleration between Charavet et al.'s study and Al-Ibrahim et al.'s study may be attributed to the difference in the systems of self-ligating brackets used (passive versus interactive in Charavet et al.'s study and Al-Ibrahim et al.'s study, respectively). The use of active self-ligating brackets in the study of Al-Ibrahim et al. may have provided better control of the movement of the anterior teeth in the leveling and alignment phase in the cases of dental crowding included in both studies [25].

Regarding the six studies that evaluated the effect of participation of self-ligating brackets with physical methods, low-level laser and low-level light combined with self-ligating brackets were effective in accelerating the orthodontic movement [35,36,38,40]. In contrast, the vibrational forces had no positive effect [34,37].

The rate of acceleration with low-level laser ranged between 20% and 50% (50%, 37.5%, and 20% in Qamruddin, Lalnunpuii, and Chandran studies, respectively) [36,38,40]. The variances can explicate these differences in the length and energy of the laser beam and the application protocols.

The 22% acceleration rate when using infrared light can be explained by the biological effects of low-level light [35]. Red and infrared lights are considered the most effective irradiation because hemoglobin does not absorb light in this range, and therefore the light can infiltrate deep into the living tissues and induce photobiomodulation [34,37]. The cytochrome oxidase and the mitochondrial enzyme, which are involved in the production of ATP, are upregulated by infrared light [34,37]. Any localized rise in ATP levels causes cells to undergo a remodeling process due to increased metabolic activity and promote their division and proliferation, which will help speed up tooth movement [34,37].

The lack of acceleration in the two studies applying low-intensity vibrational forces could be explained by the fact that the mechanical stimulation released by these forces is weak and unable to activate bone remodeling. The only effect of these forces was only IL-1β levels increasing, which had no impact on the rate of orthodontic movement.

All three studies that evaluated the status of periodontal tissues did not find any side effects following the studied therapeutic interventions (flapless corticotomy and vibration forces) [25,33,34]. This could be elucidated by the nature of evaluated surgical or physical interventions, which were not invasive.

Limitations of the current review

About three-quarters of the included studies were not at low risk of bias (two studies had some aspects of bias, and three were at high risk of bias). Thus, more low-risk of bias randomized controlled clinical trials are required to evaluate the effectiveness of combining self-ligated brackets with different acceleration methods. Five of eight studies did not evaluate the side effects of applying acceleration methods. Moreover, the side effects studied in the remaining three studies were not comprehensive, and therefore it was difficult to give a clear assessment regarding side effects due to the lack of available information. Most studies did not assess the entire duration of the studied orthodontic treatment. Long-term follow-up of the studied interventions was also absent in all included trials.

## Conclusions

The combination between self-ligating brackets and flapless corticotomy, low-level laser, or infrared light effectively accelerated the orthodontic movement by 20% to 50%. In contrast, the combination of self-ligating brackets with vibrational forces had no effect on speeding tooth movement. The acceleration methods used did not have any side effects on the periodontal tissues, but the available evidence was insufficient. There is a need for further primary research regarding the effectiveness of combining self-ligating brackets with acceleration methods, as well as the consequent side effects.
